# Visceral Fat Is a Negative Determinant of Bone Health in Obese Postmenopausal Women

**DOI:** 10.3390/ijerph17113996

**Published:** 2020-06-04

**Authors:** Deepti K. Sharma, Paul H. Anderson, Howard A. Morris, Peter M. Clifton

**Affiliations:** Clinical and Health Sciences Academic Unit, University of South Australia, Adelaide 5001, Australia; deepti.sharma@unisa.edu.au (D.K.S.); peter.clifton@unisa.edu.au (P.M.C.)

**Keywords:** visceral fat, CTX-1, bone turnover, obesity, postmenopausal women, parathyroid hormone, vitamin D

## Abstract

The protective effect of obesity on bone health has been challenged by studies that link visceral adiposity to poor bone microarchitecture in young obese men and women. In postmenopausal women, the role of visceral adipose tissue (VAT) on bone turnover markers (BTMs) has not been investigated. The aim was to investigate the impact of VAT on BTMs, total bone mineral density (BMD), vitamin D metabolites and parathyroid levels (1-84 PTH) levels in postmenopausal women. A total of 76 lean and overweight women (without osteoporosis) underwent VAT measurements by dual-energy X-ray absorptiometry (iDXA). Blood samples were analyzed for serum C-terminal telopeptide of type 1 collagen (CTX-1), osteocalcin, bone-specific alkaline phosphatase (bone ALP), 1–84 PTH and vitamin D (25 hydroxyvitamin D, 25(OH)D) levels. VAT volumes ranged from 91 to 3392 cm^3^ and body mass index (BMI) ranged from 18.3 to 53.9 kg/m^2^. Women in the highest VAT quartile had significantly lower CTX-1, 25(OH)D, osteocalcin and the highest BMD (*p* < 0.05, for all). While VAT positively associated with BMD, after controlling for BMI, VAT was a negative predictor of BMD (β = 0.368, *p* < 0.05). VAT was an independent negative predictor of CTX-1 (β = −0.263, *p* < 0.05) and osteocalcin levels (β = −0.277, *p* < 0.05). Among all measures of adiposity, VAT was the strongest independent determinant of BMD and BTMs. In clinical settings, VAT, and not BMI, may be a sensitive predictor of bone health in obese women.

## 1. Introduction

The impact of obesity on bone metabolism and risk of fractures has been of major interest, with considerable controversy and debate. Previously, obesity, when measured as body mass index (BMI), was thought to be protective for bone health, as individuals with a higher BMI usually have a higher bone mineral density (BMD) [1]. However, the risk of fractures is not low in this population, with several studies demonstrating that obesity, when measured as total and regional adipose mass, is associated with a higher risk of some site-specific fractures [2,3,4,5]. Furthermore, meta-analyses of prospective studies suggest that a 0.1 unit increase in the waist-hip ratio increased the risk of hip fracture by 3% [6], indicating that higher central or abdominal fat is associated with a higher risk of hip fracture. Consistent with this, studies using high resolution computed tomography (CT) imaging demonstrate that visceral adipose tissue (VAT) is more strongly associated with poor bone microarchitecture and reduced bone strength than other fat regions [7,8,9,10]. Abdominal subcutaneous adipose tissue (SAT) is reported to have no [11] or even protective effects on bone density and strength [8]. Bredella et al. have reported in two separate cross-sectional studies that abdominal adiposity was a negative predictor of microarchitecture and mechanical properties in young obese men [9] and a negative predictor of lumbar spine BMD in premenopausal women [10]. In addition, healthy premenopausal women with highest tertile of trunk fat levels, which approximates to VAT, exhibited inferior bone quality and stiffness, as well as reduced bone formation in transiliac bone biopsies despite reporting normal BMD [7]. These studies, which suggest that higher amount of VAT is detrimental to bone structure and strength, are consistent with reports of the harmful effect of higher VAT even when BMI is within the normal range [8]. Even when longitudinal studies associate higher VAT with higher BMD and improved microarchitecture [12], when these associations were adjusted for BMI, the association between VAT and microarchitecture were no longer significant, suggesting that effects of VAT on the skeleton were mediated by mechanical loading (weight) rather than metabolic effects of VAT. This is consistent with findings reported in a recent Mendelian randomization study in 594 postmenopausal Korean women, which showed that abdominal adiposity (defined as BMI-adjusted waist circumference, a surrogate for VAT) was neutral to BMD in postmenopausal women [13]. The possible mechanisms by which VAT mediates a detrimental effect on bones are not fully elucidated. Some investigators have shown that higher VAT is associated with lower bone formation markers and reduced insulin-like growth factor 1 (IGF-1) levels in premenopausal women [7,10] and with decreased growth hormone and testosterone levels in men [9].

To the best of our knowledge, none of the studies so far have investigated the effects of abdominal VAT and SAT on bone turnover markers (BTM) in obese postmenopausal women. A study in Egyptian obese postmenopausal investigated the effect of BMI on BMD, leptin and urinary C-terminal telopeptide of type 1 collagen (CTX-1) levels [14] and reported that women with higher BMI (>30 kg/m^2^) had significantly higher CTX-1 levels and lower hip and spine BMD when compared to lean women (BMI < 25 kg/m^2^). The aim of the present study was to investigate the effect of abdominal fat, measured as SAT and VAT levels with BTMs, total BMD, vitamin D (25-hydroxyvitamin D, 25(OH)D), 1,25-dihydroxyvitamin D3 (1,25(OH)_2_D) levels and parathyroid hormone (1-84 PTH) levels in postmenopausal women with varying BMI levels.

## 2. Subjects and Methods 

### 2.1. Participant Recruitment

Seventy-six normal (BMI < 25 kg/m^2^, *n* = 27) and overweight (BMI > 27 kg/m^2^, *n* = 50) postmenopausal women with no osteoporosis, no history of low trauma fractures and no causes of potential bone loss were selected for the study. The women were recruited through advertisements placed in the local newspapers, social media and radio between July–November 2017. The aim was to recruit women with varying levels of VAT. As BMI is the most widely used categorization system for obesity, we recruited women in two BMI groups; less than 25 kg/m^2^ and greater than 27 kg/m^2^. This criterion allowed us to recruit women with VAT levels distributed over a range (91 to 3392 cm^3^). The inclusion criteria were women who were at least 5 years postmenopausal, not currently taking calcium or vitamin D supplements, or, if so, agreeing to stop these for 4 weeks before entering the trial. The exclusion criteria were women taking any medications known to affect bone metabolism, diabetes or a fracture in the past 12 months. Institutional ethics approval was obtained, and the trial was registered at the Australia New Zealand Clinical Trial Registry (ACTRN12617000779370).

### 2.2. BMD and Body Composition Analyses 

All participants underwent body weight, hip and waist circumference (WC) measurements. Total body dual-energy X-ray absorptiometry scans were acquired (Lunar iDXA, GE Healthcare, Madison, WI, USA) to measure whole-body fat mass, lean mass, android fat, gynoid fat, android:gynoid ratio, total BMD, SAT and VAT over the android region (CoreScan, GE Healthcare, Madison, WI, USA). iDXA Corescan software has been validated to measure VAT [15] and precision CV (coefficient of variation) for VAT measurement has been reported to be 5.1% [16]. 

### 2.3. Biochemical Analyses

Serum samples were analyzed by chemiluminescent immunoassays (Diasorin LiaisonXL) for 25(OH)D, 1,25(OH)_2_D, 1–84 PTH levels (3rd generation PTH assay), osteocalcin levels (a bone turnover marker) and bone-specific alkaline phosphatase (bone ALP), a bone formation marker. The precision CVs for the assays as provided by the manufacturer were: 3.8% and 0.1% (within run) and 9.8% and 9.1% (between run) for 25(OH)D at concentrations of 19.6 and 301.3 nmol/L; 2.7% and 5% (within run) and 3.8% and 3.6% (between run) for 1,25(OH)_2_D at concentrations of 30.9 and 122.9 pmol/L; 3.4% and 1.9% (within run) and 3.9% and 3.3% (between run) for bone ALP at concentrations of 6.83 and 37.9 µg/L; 12% and 4% (within run) and 14% and 6% (between run) for osteocalcin at concentrations of 10.4 and 238 ng/mL; 4.9% and 4.8% (within run) and 7.2% and 5.6% (between run) for 1–84 PTH assay at concentrations of 3 and 30.5 pmol/L. 

Serum calcium, phosphorus and alkaline phosphatase (ALP) levels were measured by colorimetric assays as per manufacturer’s instructions (Kone lab 20, Thermo Fisher, NSW, Australia). The human reference range is 2.1–2.55 mmol/L for calcium, 0.65–1.45 mmol/L for phosphorus and 30–110 U/L for alkaline phosphate. The 95% observed range for the assays were 47.76–190.32 pmol/L for 1,25(OH)_2_D, 0.69–3.9 pmol/L for 1–84 PTH, 0.92–10.11 nmol/L for osteocalcin, and 5.2–24.4 µg/L for bone ALP in postmenopausal women.

Plasma CTX-1 were measured by 2-step chemiluminescence immunoassay (IDS-iSYS, Immunodiagnostic Systems) as per the recommendation from International Osteoporosis Foundation (IOF)-International Federation of Clinical Chemistry and Laboratory Medicine (IFCC) Joint Working Group on Bone Marker Standards (WG-BMS) [17,18]. The precision CVs as provided by the manufacturer at 216 ng/L, 877 ng/L and 2270 ng/L are 4.9%, 2.1% and 2.4% respectively within run and 8.8%, 5.1% and 4.7 % respectively between run. Mean CTX-1 and 1–84 PTH levels were obtained from independent measurements of fasting blood specimens taken on three different days.

### 2.4. Sample Size and Statistical Analyses

Normality was assessed with the use of histograms and Shapiro–Wilk test. Most of the variables (BMD, BMI, WC, CTX-1 levels, bone ALP levels, 1,25(OH)_2_D levels, 1–84 PTH levels) were not normally distributed; hence, Spearman coefficient of correlation was used to describe the relationships between body fat variables and bone markers. To investigate the effect of VAT on bone health, participants were divided into VAT quartiles and nonparametric Kruskal–Wallis test was used to compare the quartiles. Multiple linear regression models were used to investigate determinants of bone and biochemical variables. All the analyses were carried out using SPSS (SPSS Stats 25, IBM, Armonk, NY, USA) and *p* < 0.05 was considered significant. With 19 participants in each group (SD 40%), we have 80% power to detect a difference between VAT Q1 and Q4 in baseline CTX-1 of 36% (unpaired *t* test) with alpha = 0.05.

## 3. Results

### 3.1. Participant Characteristics

The median age of the participants was 65.5 year of age (interquartile range 61.9–68.5 years). An amount of 36% women had normal BMI (<24.9 kg/m^2^, *n* = 27), 29% women were overweight (BMI 27–29.9 kg/m^2^, *n* = 22) and 36% were obese (>30 kg/m^2^, *n* = 27). Median abdominal fat was 49% and gynoid fat was 47%. Median VAT volume was 946 cm^3^, SAT volume was 1780 cm^3^ and CTX-1 levels were 417 ng/L. The amount of VAT varied significantly even within a narrow BMI range. VAT volumes in the lean group (BMI < 25 kg/m^2^) ranged from 91–1050 cm^3^ and in the overweight group (BMI > 27 kg/m^2^) ranged from 425–3392 cm^3^. Hence, to investigate the effect of VAT, participants were divided into VAT quartiles and their characteristics and biochemical profiles as per VAT quartiles are described in Table 1 and Table 2, respectively. There was no difference in participant’s age across quartiles, BMI was normal in the first quartile (<25 kg/m^2^) and all measures of adiposity such as BMI, WC, android fat and SAT as well as BMD increased with increasing VAT volume. VAT and WC correlated positively and significantly (r = 0.855, *p* < 0.001). The median WC was less than <88 cm—the recommended cut-off reference [19] in the first and second quartile—and increased significantly across the third and fourth quartile. The biochemical profile for all the study participants were within the reference range, though the levels for CTX-1, vitamin D, osteocalcin and 1-84 PTH varied significantly between the VAT quartiles (Table 2).

### 3.2. Spearman Correlations between Bone Markers, Body Fat and Metabolic Variables

**Figure 1:**
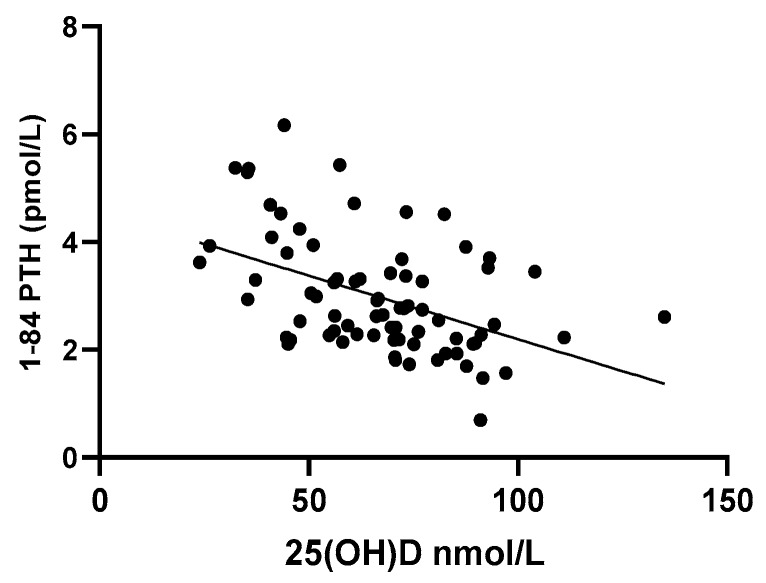
Linear regression analyses between vitamin D (25(OH)D) and parathyroid hormone (1–84 PTH) levels.

Osteocalcin levels and bone ALP levels correlated positively with CTX-1 levels (r = 0.679, *p* < 0.001 and r = 0.401, *p* < 0.001, respectively). Osteocalcin and bone ALP levels also correlated positively with each other (r = 0.235, *p* = 0.041). None of the bone markers correlated with 25(OH)D or 1–84 PTH levels, but a positive correlation with 1,25(OH)_2_D was observed for CTX-1 (r = 0.370, *p* = 0.001) and osteocalcin (r = 0.305, *p* = 0.007) levels. A total of 25(OH)D levels correlated negatively with 1–84 PTH levels (r = −0.43, *p* < 0.001; Figure 1). Circulating 1,25(OH)_2_D levels correlated negatively with BMD (r = −0.299, *p* = 0.009) and positively with CTX-1 levels (r = 0.370, *p* = 0.001) and osteocalcin (r = 0.305, *p* = 0.007).

The correlations between body fat measures and bone markers are presented in Table 3. CTX-1 and osteocalcin levels corelated negatively with BMI, WC, VAT and SAT but no correlation with gynoid fat was observed. Bone ALP levels showed no association with body fat variables (Table 3). No association was observed between whole body lean mass and bone markers.

### 3.3. Effect VAT on CTX-1, Osteocalcin and Bone ALP Levels

Women in the highest VAT quartile had significantly lower CTX-1 and osteocalcin levels (*p* < 0.05 for both, Table 2). Bone ALP levels were similar across VAT quartiles. CTX-1 and osteocalcin levels correlated negatively with BMI, WC, SAT and VAT levels, while no correlation was observed between bone ALP levels and any body composition variable. To investigate the independent body fat predictors of CTX-1 and osteocalcin levels, step-wise multiple linear regression analyses was performed with BMI, VAT, SAT and WC as independent variables. VAT levels were the only significant body fat predictor of CTX-1 levels (β = −0.302, *p* = 0.008) and osteocalcin levels (β = −0.306, *p* = 0.007). Serum 1,25(OH)_2_D levels accounted for 23% of variability in CTX-1 levels (Table 4) and 17% variability in osteocalcin levels (Table 5), as determined by stepwise modeling (Table 5). 

### 3.4. Effect of VAT on total BMD

BMD values increased across VAT quartiles (Table 1). BMD was positively associated with all body composition measures: BMI, r = 0.6; whole body lean mass, r = 0.5; whole body fat mass, r = 0.568; WC, r = 0.42; VAT, r = 0.432; abdominal SAT, r = 0.551; and gynoid fat mass, r = 0.414. *p* < 0.001 for all. Among the metabolic markers, BMD was negatively associated with CTX-1 (r = −0.357, *p* < 0.05, osteocalcin (r = −0.226, *p* = 0.05) and 1,25(OH)_2_D (r = −0.299, *p* < 0.05) levels while no association between bone ALP levels and BMD was found. 

Abdominal SAT and VAT levels accounted for 30% and 18% variability respectively in BMD in univariate analyses. To investigate the independent predictors of BMD, multiple linear regression analyses was carried out with BMI, lean mass, VAT, SAT, WC, gynoid fat, CTX-1, osteocalcin and 1,25(OH)_2_D levels as independent variables. BMI, CTX-1 and VAT levels were the only significant predictors of BMD and together accounted for 48% variability in BMD (*p* < 0.001, Table 6). While VAT correlated positively with BMD, it was a negative predictor of BMD when controlled for BMI (β = −0.368, *p* = 0.017). No influence of collinearity between BMI and fat variables occurred with the variance inflation factor (VIF) less than 3.5 [20,21].

### 3.5. Effect of VAT on Vitamin D Metabolites 

The median 25(OH)D levels were 68.6 nmol/L (range 24–135 nmol/L) and the levels decreased across VAT quartiles (*p* < 0.05, Table 2). Women in the highest VAT quartile (Q4) had the lowest 25(OH)D levels. Serum 25(OH)D levels were significantly and negatively associated with measures of total adiposity and central fat (except WC, *p* = 0.052, Table 3). Abdominal SAT and VAT accounted for 11% and 6% variance respectively in 25(OH)D levels. The median 1,25(OH)_2_D levels in this cohort were 119 pmol/L (range 76–220 pmol/L) and the levels were similar across VAT quartiles. The 1,25(OH)_2_D levels were not associated with any of the body composition variables. 

### 3.6. Effect of VAT on PTH Levels

The median 1–84 PTH levels in this cohort were 2.63 pmol/L (range 0.57–6.02 pmol/L). A total of 13% of the women had elevated 1–84 PTH levels (>3.9 pmol/L). The 1–84 PTH concentration was significantly different between VAT quartiles (*p* = 0.002) and women with highest VAT (Q4) had the highest 1–84 PTH levels. Serum 1–84 PTH levels were positively associated with all measures of body fat (Table 3). Abdominal SAT and VAT accounted for 11% and 13% variability, respectively, in 1–84 PTH levels in univariate analyses. Stepwise multiple regression modeling was conducted to investigate predictors of 1–84 PTH levels with BMI, VAT, SAT, gynoid fat, WC, total calcium and 25(OH)D entered as independent variables. VAT and 25(OH)D levels together accounted for 24% variability in 1–84 PTH (*p* < 0.001) and the rest of the variables were removed from the model (Table 7). 

### 3.7. Effect of VAT on Calcium, ALP and Phosphorus

All participants had normal serum calcium (2.35 mmol/L, IQ range 2.29–2.39), phosphorus (1.17 mmol/L, IQ range 1.09–1.22) and alkaline phosphatase (ALP) (63.97 U/L, IQ range 55.59–77.71 U/L). There was no correlation between body fat measures and calcium and the levels were similar across VAT quartiles. Phosphorus levels were negatively associated with BMI, VAT and WC (Table 3) but the levels were not significantly different across VAT quartiles (Table 2). ALP levels were positively associated with abdominal SAT and VAT levels (Table 3). 

## 4. Discussion

This is the first study to investigate the effect of abdominal fat measured as SAT and VAT on bone markers, total BMD, 25(OH)D and 1–84 PTH levels in postmenopausal women. Postmenopausal women are generally in a high state of bone turnover due to estrogen deficiency with a 79–97% increase in bone resorption rate [22]. However, the present study shows that obese postmenopausal women exhibit a state of suppressed bone turnover with reduced CTX-1 and osteocalcin levels. Furthermore, only VAT levels independently determined lower bone turnover despite also being associated with elevated serum 1–84 PTH levels. Interestingly, while a positive correlation occurred between VAT and BMD, this was determined by BMI levels, as after controlling for BMI, VAT levels were a negative predictor of BMD, consistent with findings in previous studies [8,10,23]. This suggests that BMI does not adequately account for variations in body fat distribution and abdominal fat mass, which can differ greatly across populations and can vary substantially within a narrow range of BMI [19]. While WC is the most widely used indicator of VAT in clinical settings [24], WC did not contribute to the strong VAT-dependent determination of these bone variables. 

The findings of reduced CTX-1 and osteocalcin levels with higher VAT levels are consistent with reports of reduced bone resorption and turnover in premenopausal women with higher trunk fat [7,10]. Lower bone resorption is possibly the cause of higher BMD observed in women with higher VAT. Furthermore, this observation is consistent with reports of reduced CTX-1 and osteocalcin levels in overweight men and women while procollagen type I N propeptide (P1NP) bone formation markers and bone ALP levels were similar across BMI groups [25]. Although a higher urinary CTX-1 levels have been reported previously in obese Egyptian postmenopausal women with BMI > 30 kg/m^2^ [14], differences in CTX-1 assays [18,26] and the absence of a urinary creatinine adjustment for urinary CTX-1 levels as per guidelines for standardized bone assays [18] make biological comparisons difficult to assess. Furthermore, this study investigated the effect of BMI and not fat distribution on bone variables. While osteocalcin is produced by osteoblasts, it is not a considered as an accurate bone formation marker since osteocalcin can also be liberated from the bone matrix during bone resorption processes [27].The reasons for reduced bone turnover in postmenopausal women with higher VAT levels is not fully understood. Reduced osteocalcin levels, which have been previously associated with insulin resistance and increased risk for diabetes [28,29,30,31], may in fact contribute to changes in VAT levels, as mice lacking osteocalcin exhibit higher VAT levels as well as glucose intolerance and impaired insulin secretion [32]. Whether lower osteocalcin levels may cause VAT accumulation in humans and whether this results in insulin resistance warrants further investigations. Reduced CTX-1 levels in postmenopausal women with higher VAT levels despite elevated 1–84 PTH levels suggest that factors other than PTH play a greater role in determining bone resorption under these circumstances. In addition, reduced bone turnover (lower osteocalcin levels) with no difference in bone ALP levels between women with higher and lower VAT suggest that lower bone turnover may not be coupled to a similar lowering of mineralization (indicated by no difference in bone ALP levels) and the matrix may become hyper-mineralized [33]. Hyper-mineralization may result in a poor microarchitecture which might be one of the reasons for higher fracture risk in obese individuals despite normal or higher BMD compared to lean individuals. Furthermore, a lower bone turnover and resorption in women with higher VAT may result in accumulation of unrepaired microdamage resulting from repeated loads applied daily to the bone which further results in lower quality bone. Visceral adiposity may also influence bone turnover via the opioid system [34] or the endocanniboid system [35] and their receptors. Both opioid [36] and endocannabinoid systems [37] are known to play a role in the regulation of food intake and thermogenesis and the metabolic alterations associated with obesity.

Lower 25(OH)D levels observed in postmenopausal women with higher VAT levels are consistent with findings in numerous studies [25,38,39,40,41]. Several reasons for lower 25(OH)D levels in obese individuals have been postulated, including sequestration of vitamin D by adipose tissue making it less bioavailable [39] or lower cutaneous synthesis with age [42]. However, most of the conclusive evidence suggests that a greater volume of distribution in obese individuals results in lower 25(OH)D levels [25,43]. Accordingly, the effects of dietary vitamin D supplementation have been found to be BMI-dependent [44,45]; hence, dietary vitamin D therapy needs to be adjusted for body size to achieve the desired serum 25(OH)D concentrations and to suppress PTH levels. The Endocrine Society Clinical Practice Guideline also suggest that 2–3-times higher vitamin D doses are required in obese subjects [46]. Despite this, the lower 25(OH)D levels in the majority of obese women were still above recommended levels for bone health [47], which would explain why no correlations were observed between vitamin D and BMD or bone turnover markers. However, the inverse association between 25(OH)D and 1–84 PTH levels coupled with the fact that VAT and 25(OH)D levels together account for 24% variability in 1–84 PTH levels raises the notion that in clinical settings, higher dietary intake of 25(OH)D may be required to suppress PTH levels in obese postmenopausal women. A negative association between 1,25(OH)_2_D levels and BMD and a positive correlation between CTX-1 and osteocalcin levels suggests that higher circulating 1,25(OH)_2_D levels may be determinantal to bone health, independent of body fat measures. This is consistent with the role of 1,25(OH)_2_D on the RANKL-signaling pathway of osteoclastogenesis [48,49,50].

This study provides data on the effect of VAT on bone turnover in a group of lean and obese postmenopausal women, which is an important and at-risk population for osteoporosis. Precise measurement of VAT using iDXA allowed us to segregate the effects of abdominal SAT from VAT and to investigate the independent associations of these fat fractions with bone turnover. However, the study has few limitations. The cross-sectional nature of the analyses does not prove causality between VAT and bone health. Furthermore, we did not measure site-specific BMD. It would be more informative to generate site-specific BMD data in postmenopausal women with higher VAT to predict fracture risk with increasing VAT. A larger study including higher numbers and an assessment of bone microarchitecture using sophisticated imaging techniques such as high resolution peripheral computed tomography (HR-pQCT) would provide greater insights on the effect of VAT on bone health at a population level.

## 5. Conclusions

While obesity is associated with increased BMD in postmenopausal women, higher visceral adipose tissue levels, independent of BMI, are detrimental to bone health. Understanding the role that visceral adipose tissue has on PTH, and on 25(OH)D levels as well as on bone turnover may provide insights into the determinants of bone health and may influence antiresorptive treatment strategies and nutritional guidelines, such as calcium supplementation for obese postmenopausal women. Furthermore, the interaction between VAT and bone turnover markers observed in this study suggests that future studies investigating the cross-talk between metabolic markers associated with higher VAT such as leptin, adiponectin, insulin resistance, inflammatory cytokines [51] and bone biomarkers and microarchitecture in women with low and high VAT would help to explain the possible links between obesity, poor bone microarchitecture and higher fracture risk.

## 6. Study Highlights

- Postmenopausal women with higher visceral fat have reduced bone resorption (CTX-1 levels) despite elevated PTH levels.
- Visceral fat is an independent negative predictor of total bone density, CTX-1 and osteocalcin levels.
- The clinical implications of low bone turnover with higher VAT levels are discussed briefly.

## Figures and Tables

**Table 1:** Participant characteristics by VAT quartiles; median (interquartile range), *n* = 19/quartile.

VAT cm^3^	Quartile 1 231 (190–425)	Quartile 2 780 (690–874)	Quartile 3 1208 (1050–1302)	Quartile 4 1723 (1615–2226)	*p*-Value <0.0001
Age, y	65.7 (61.8–68.8)	65.4 (61.4–68.1)	66.3 (64.2–69)	64.8 (61.2–67.3)	0.4 (NS)
Weight, cm	57.7 (48.2–63.8)	68.5 (62.7–78)	74.8 (72.1–81)	93 (83.3–100.2)	<0.0001
Height, cm	159.4 (155.1–162.6)	165.6 (156.8–169.5)	163.3 (157.4–165.8)	161.6 (158.2–164.5)	0.15 (NS)
BMI, kg/m^2^	22.9 (20.2–24.0)	27.3 (24.0–27.9)	28.0 (27.2–29.5)	35.5 (32.0–39.5)	<0.0001
WC, cm	75 (71–77.5)	85.6 (81.5–94.7)	93.5 (88–96)	109.5 (102–118)	<0.0001
Total fat mass, kg	18.9 (13.0–22.3)	28.1 (24.1–31.2)	32.6 (29.1–36.5)	44.9 (37.9–49.8)	<0.0001
Total lean mass, kg	3.5 (3.3–3.9)	39.7 (34.5–43.4)	38.6 (36.3–41.2)	45.1 (39.7–48.6)	<0.0001
Android fat mass, kg	4.1 (3.4–4.4)	5 (4.7–5.9)	5.8 (5.3–6.1)	7.6 (6.8–8.8)	<0.0001
Android lean mass, kg	2.7 (2.5–2.9)	2.9 (2.6–3.2)	2.7 (2.5–3.2)	3.3 (2.9–3.9)	<0.0001
Android fat mass, kg	1.4 (0.8–1.5)	2.3 (1.9–2.6)	2.9 (2.6–3.2)	4.2 (3.8–4.8)	<0.0001
Android fat (%)	33.7 (24.8–36.9)	44.8 (42.2–49.9)	52.1 (46.4–54.2)	56.5 (54.7–58.8)	<0.0001
Gynoid fat (%)	39.5 (33.8–43.8)	47.4 (42.6–48.8)	47.8 (44.1–49.9)	50.1 (48.2–53.1)	<0.0001
SAT, cm^3^	1178 (759–1407)	1714 (1321–2107)	1817 (1605–2178)	2510 (2138–3376)	<0.0001
Android/Gynoid ratio	0.88 (0.74–0.94)	1.0 (0.9–1.04)	1.1 (1.0–1.17)	1.13 (1.08–1.17)	<0.0001
BMD, g/cm^2^	1.04 (0.91–1.14)	1.11 (0.99–1.14)	1.12 (0.99–1.2)	1.17 (1.10–1.23)	0.016

Notes. VAT: visceral adipose tissue; BMI: body mass index; WC: waist circumference; SAT: subcutaneous adipose tissue; BMD: bone mineral density.

**Table 2:** Participant biochemical profile by VAT quartiles; median (interquartile range) *n* = 19/quartile.

VAT cm^3^	Quartile 1 231 (190–425)	Quartile 2 780 (690–874)	Quartile 3 1208 (1050–1302)	Quartile 4 1723 (1615–2226)	*p*-Value <0.0001
Calcium mmol/L	2.34 (2.28–2.41)	2.35 (2.32–2.38)	2.36 (2.26–2.41)	2.33 (2.29–2.37)	0.9
Phosphorus mmol/L	1.21 (1.09–1.23)	1.18 (1.11–1.27)	1.16 (1.14–1.23)	1.08 (0.96–1.21)	0.2
ALP U/L	56.6 (48.7–68.2)	69.4 (57.6–74.8)	60.9 (54.6–89.4)	77.7 (60.8–91.6)	0.007
25 (OH)D nmol/L	72.2 (61.5–91.6)	67.7 (47.8–77.1)	71.5 (51–80.8)	57.4 (41.1–73.2)	0.029
1,25 (OH)_2_D pmol/L	126 (107–157)	126 (102–141)	116 (98.5–154)	117 (106–138)	0.767
1–84 PTH pmol/L	2.4 (2.2–2.6)	2.5 (2.1–3.5)	2.8 (2.2–3.5)	3.4 (3.0–4.1)	0.002
Osteocalcin nmol/L	3.8 (3.4–4.4)	4.1 (3.5–4.5)	3.6 (2.9–4.4)	3.3 (2.9–3.5)	0.025
Bone ALP µg/L	11.6 (9.4–13.1)	13.1 (11.1–15.8)	12.4 (9.2–17.3)	14.1 (9.6–19.6)	0.378
CTX-1 ng/L	454.2 (397.5–640.6)	476.5 (346.2–701.2)	402.4 (320.5–615.7)	314 (248–418)	0.041

Notes. VAT: visceral adipose tissue; ALP: alkaline phosphatase; 25(OH)D: vitamin D or 25-hydroxyvitamin D; 1,25(OH)_2_D: 1,25-dihydroxyvitamin D3; 1-84 PTH: parathyroid hormone; bone ALP: bone-specific alkaline phosphatase; CTX-1: C-terminal telopeptide of type I collagen.

**Table 3:** Spearman correlation between body fat measures and metabolic markers.

Variables		25(OH)D nmol/l	1–84 PTH pmol/l	Osteocalcin nmol/l	CTX-1 ng/l	Bone ALP µg/l	ALP U/L	Calcium mmol/l	Phosphorus mmol/l
BMI	r	−0.30	0.39	−0.31	−0.32	0.16	0.32	0.018	−0.28
	*p*	0.008	0.001	0.007	0.005	NS	0.004	NS	0.014
WC (cm)	r	−0.22	0.33	−0.30	−0.29	0.086	0.24	0.07	−0.28
	*p*	NS	0.003	0.008	0.010	NS	0.038	NS	0.016
VAT (cm^3^)	r	−0.24	0.37	−0.35	−0.36	0.18	0.37	0.002	−0.24
	*p*	0.034	0.001	0.002	0.002	NS	0.001	NS	0.041
SAT (cm^3^)	r	−0.34	0.36	−0.23	−0.26	0.20	0.32	−0.05	−0.17
	*p*	0.003	0.002	0.042	0.025	NS	0.004	NS	NS
Gynoid fat (%)	r	−0.24	0.3	−0.16	−0.20	0.13	0.22	−0.05	−0.28
	*p*	0.035	0.008	NS	0.076	NS	0.06	NS	0.014

Notes. 25(OH)D: vitamin D or 25-hydroxyvitamin D; 1-84 PTH: parathyriod hormone; CTX-1: C-terminal telopeptide of type I collagen; Bone ALP: bone-specific alkaline phophatase; ALP: alkaline phophatase; WC: waist circumference; VAT: visceral adipose tissue; SAT: subcutaneous adipose tissue; NS: non-significant).

**Table 4:** Determinants of C-terminal telopeptide of type I collagen (CTX-1) levels—stepwise multivariate regression.

Independent Predictors	Standardized Coefficient (β)	t	*p*
1,25(OH)_2_D	0.404	3.980	<0.001
VAT	−0.263	−2.591	0.012
Constant	193	1.932	0.057

Independent variables added: body mass index (BMI), visceral adipose tissue (VAT), subcutaneous adipose tissue (SAT), waist circumference (WC) and 1,25-dihydroxyvitamin D3 (1,25(OH)_2_D).

**Table 5:** Determinants of osteocalcin levels—stepwise multivariate regression.

Independent Predictors	Standardized Coefficient (β)	t	*p*
1,25(OH)_2_D	0.311	2.940	0.004
VAT	−0.277	−2.617	0.011
Constant	2.89	6.341	<0.001

Independent variables added: body mass index (BMI), visceral adipose tissue (VAT), subcutaneous adipose tissue (SAT), waist circumference (WC) and 1,25-dihydroxyvitamin D3 (1,25(OH)_2_D).

**Table 6:** Determinants of BMD—stepwise multivariate regression.

Independent Predictors	Standardized Coefficient (β)	t	*p*
BMI	0.871	5.833	<0.001
CTX-1	−0.279	−3.198	0.002
VAT	−0.368	−2.435	0.017
Constant	0.764	11.383	<0.001

Independent variables added: body mass index (BMI), lean mass, visceral adipose tissue (VAT), subcutaneous adipose tissue (SAT), waist circumference (WC), gynoid fat, C-terminal telopeptide of type I collagen (CTX-1), osteocalcin and 1,25-dihydroxyvitamin D3 (1,25(OH)_2_D).

**Table 7:** Determinants of 1–84 PTH levels—stepwise multivariate regression.

Independent Predictors	Standardized Coefficient (β)	t	*p*
25(OH)D	−0.361	−3.455	0.001
VAT	0.274	2.620	0.011
Constant	3.727	8.501	<0.001

Independent variables added: body mass index (BMI), visceral adipose tissue (VAT), subcutaneous adipose tissue (SAT), percent gynoid fat, waist circumference (WC), total calcium and vitamin D (25(OH)D).

## References

[1] Evans A.L., Paggiosi M.A., Eastell R., Walsh J.S. (2015). Bone density, microstructure and strength in obese and normal weight men and women in younger and older adulthood. J. Bone Miner. Res..

[2] Compston J.E., Watts N.B., Chapurlat R., Cooper C., Boonen S., Greenspan S., Pfeilschifter J., Silverman S., Diez-Perez A., Lindsay R. (2011). Obesity is not protective against fracture in postmenopausal women: GLOW. Am. J. Med..

[3] Prieto-Alhambra D., Premaor M.O., Fina Avilés F., Hermosilla E., Martinez-Laguna D., Carbonell-Abella C., Nogués X., Compston J.E., Díez-Pérez A. (2012). The association between fracture and obesity is site-dependent: A population-based study in postmenopausal women. J. Bone Miner. Res..

[4] Johansson H., Kanis J.A., Oden A., McCloskey E., Chapurlat R.D., Christiansen C., Cummings S.R., Diez-Perez A., Eisman J.A., Fujiwara S. (2014). A meta-analysis of the association of fracture risk and body mass index in women. J. Bone Miner. Res..

[5] Beck T.J., Petit M.A., Wu G., LeBoff M.S., Cauley J.A., Chen Z. (2009). Does Obesity Really Make the Femur Stronger? BMD, Geometry, and Fracture Incidence in the Women’s Health Initiative-Observational Study. J. Bone Miner. Res..

[6] Li X., Gong X., Jiang W. (2017). Abdominal obesity and risk of hip fracture: A meta-analysis of prospective studies. Osteoporos Int..

[7] Cohen A., Dempster D.W., Recker R.R., Lappe J.M., Zhou H., Zwahlen A., Müller R., Zhao B., Guo X., Lang T. (2013). Abdominal Fat Is Associated With Lower Bone Formation and Inferior Bone Quality in Healthy Premenopausal Women: A Transiliac Bone Biopsy Study. J. C. Endocrinol. Metab..

[8] Gilsanz V., Chalfant J., Mo A.O., Lee D.C., Dorey F.J., Mittelman S.D. (2009). Reciprocal relations of subcutaneous and visceral fat to bone structure and strength. J. Clin. Endocrinol. Metab..

[9] Bredella M.A., Lin E., Gerweck A.V., Landa M.G., Thomas B.J., Torriani M., Bouxsein M.L., Miller K.K. (2012). Determinants of bone microarchitecture and mechanical properties in obese men. J. Clin. Endocrinol. Metab..

[10] Bredella M.A., Torriani M., Ghomi R.H., Thomas B.J., Brick D.J., Gerweck A.V., Harrington L.M., Breggia A., Rosen C.J., Miller K.K. (2011). Determinants of bone mineral density in obese premenopausal women. Bone.

[11] Hind K., Pearce M., Birrell F. (2017). Total and Visceral Adiposity Are Associated With Prevalent Vertebral Fracture in Women but Not Men at Age 62 Years: The Newcastle Thousand Families Study. J. Bone Miner. Res..

[12] Liu C.T., Broe K.E., Zhou Y., Boyd S.K., Cupples L.A., Hannan M.T., Lim E., McLean R.R., Samelson E.J., Bouxsein M.L. (2017). Visceral Adipose Tissue Is Associated With Bone Microarchitecture in the Framingham Osteoporosis Study. J. Bone Miner. Res..

[13] Lee S.J., Lee J.Y., Sung J. (2019). Obesity and Bone Health Revisited: A Mendelian Randomization Study for Koreans. J. Bone Miner. Res..

[14] Ibrahim S.E., ElShishtawy H.F., Helmy A., Galal Z.A., Abdel Salam M.H. (2011). Serum leptin concentration, bone mineral density and bone biochemical markers in a sample of Egyptian women: A possible relationship. Egypt. Rheumatol..

[15] Kaul S., Rothney M.P., Peters D.M., Wacker W.K., Davis C.E., Shapiro M.D., Ergun D.L. (2012). Dual-energy X-ray absorptiometry for quantification of visceral fat. Obesity.

[16] Rothney M.P., Xia Y., Wacker W.K., Martin F.P., Beaumont M., Rezzi S., Giusti V., Ergun D.L. (2013). Precision of a new tool to measure visceral adipose tissue (VAT) using dual-energy X-Ray absorptiometry (DXA). Obesity.

[17] Morris H.A., Eastell R., Jorgensen N.R., Cavalier E., Vasikaran S., Chubb S.A.P., Kanis J.A., Cooper C., Makris K. (2017). Clinical usefulness of bone turnover marker concentrations in osteoporosis. Clin. Chim. Acta.

[18] Vasikaran S., Eastell R., Bruyère O., Foldes A.J., Garnero P., Griesmacher A., McClung M., Morris H.A., Silverman S., Trenti T. (2011). Markers of bone turnover for the prediction of fracture risk and monitoring of osteoporosis treatment: A need for international reference standards. Osteoporos. Int..

[19] Waist circumference and Waist-Hip Ratio, Report of a WHO Expert Consultation GENEVA, 8–11 December 2008. https://www.who.int/nutrition/publications/obesity/WHO_report_waistcircumference_and_waisthip_ratio/en/.

[20] Hair J.F., Black W.C., Babin B.J., Anderson R.E. (2014). Multivariate Data Analysis.

[21] Akinwande O., Dikko H.G., Agboola S. (2015). Variance Inflation Factor: As a Condition for the Inclusion of Suppressor Variable(s) in Regression Analysis. Open J. Stat..

[22] Garnero P., Sornay-Rendu E., Chapuy M.-C., Delmas P.D. (1996). Increased bone turnover in late postmenopausal women is a major determinant of osteoporosis. J. Bone Miner. Res..

[23] Choi H.S., Kim K.J., Kim K.M., Hur N.W., Rhee Y., Han D.S., Lee E.J., Lim S.K. (2010). Relationship between visceral adiposity and bone mineral density in Korean adults. Calcif. Tissue Int..

[24] Pouliot M.C., Despres J.P., Lemieux S., Moorjani S., Bouchard C., Tremblay A., Nadeau A., Lupien P.J. (1994). Waist circumference and abdominal sagittal diameter: Best simple anthropometric indexes of abdominal visceral adipose tissue accumulation and related cardiovascular risk in men and women. Am. J. Cardiol..

[25] Walsh J.S., Evans A.L., Bowles S., Naylor K.E., Jones K.S., Schoenmakers I., Jacques R.M., Eastell R. (2016). Free 25-hydroxyvitamin D is low in obesity, but there are no adverse associations with bone health. Am. J. Clin. Nutr..

[26] Huber F., Traber L., Roth H.J., Heckel V., Schmidt-Gayk H. (2003). Markers of bone resorption--measurement in serum, plasma or urine?. Clin. Lab..

[27] Cavalier E., Eastell R., Jørgensen N.R., Makris K., Vasikaran S., Morris H.A., Huhtaniemi I., Martini L. (2018). Bone Turnover Markers. Encyclopedia of Endocrine Diseases.

[28] Garcia-Martin A., Cortes-Berdonces M., Luque-Fernandez I., Rozas-Moreno P., Quesada-Charneco M., Munoz-Torres M. (2011). Osteocalcin as a marker of metabolic risk in healthy postmenopausal women. Menopause.

[29] Lee S.W., Jo H.H., Kim M.R., You Y.O., Kim J.H. (2012). Association between obesity, metabolic risks and serum osteocalcin level in postmenopausal women. Gynecol. Endocrinol..

[30] Tonks K.T., White C.P., Center J.R., Samocha-Bonet D., Greenfield J.R. (2017). Bone Turnover Is Suppressed in Insulin Resistance, Independent of Adiposity. J. Clin. Endocrinol. Metab..

[31] Viljakainen H., Ivaska K.K., Paldanius P., Lipsanen-Nyman M., Saukkonen T., Pietilainen K.H., Andersson S., Laitinen K., Makitie O. (2014). Suppressed bone turnover in obesity: A link to energy metabolism? A case-control study. J. Clin. Endocrinol. Metab..

[32] Lee N.K., Sowa H., Hinoi E., Ferron M., Ahn J.D., Confavreux C., Dacquin R., Mee P.J., McKee M.D., Jung D.Y. (2007). Endocrine regulation of energy metabolism by the skeleton. Cell.

[33] Starup-Linde J., Lykkeboe S., Gregersen S., Hauge E.M., Langdahl B.L., Handberg A., Vestergaard P. (2016). Bone Structure and Predictors of Fracture in Type 1 and Type 2 Diabetes. J. Clin. Endocrinol. Metab..

[34] Coluzzi F., Pergolizzi J., Raffa R.B., Mattia C. (2015). The unsolved case of “bone-impairing analgesics”: The endocrine effects of opioids on bone metabolism. Ther. Clin. Risk Manag..

[35] Idris A.I., Ralston S.H. (2010). Cannabinoids and Bone: Friend or Foe?. Calcif. Tissue Int..

[36] Cozzolino D., Sessa G., Salvatore T., Sasso F.C., Giugliano D., Lefebvre P.J., Torella R. (1996). The involvement of the opioid system in human obesity: A study in normal weight relatives of obese people. J. Clin. Endocrinol. Metab..

[37] Rossi F., Punzo F., Umano G.R., Argenziano M., Miraglia Del Giudice E. (2018). Role of Cannabinoids in Obesity. Int. J. Mol. Sci..

[38] Arunabh S., Pollack S., Yeh J., Aloia J.F. (2003). Body fat content and 25-hydroxyvitamin D levels in healthy women. J. Clin. Endocrinol. Metab..

[39] Wortsman J., Matsuoka L.Y., Chen T.C., Lu Z., Holick M.F. (2000). Decreased bioavailability of vitamin D in obesity. Am. J. Clin. Nutr..

[40] Saarnio E., Pekkinen M., Itkonen S.T., Kemi V., Karp H., Ivaska K.K., Risteli J., Koivula M.K., Karkkainen M., Makitie O. (2018). Low free 25-hydroxyvitamin D and high vitamin D binding protein and parathyroid hormone in obese Caucasians. A complex association with bone?. PLoS ONE.

[41] Snijder M.B., van Dam R.M., Visser M., Deeg D.J., Dekker J.M., Bouter L.M., Seidell J.C., Lips P. (2005). Adiposity in relation to vitamin D status and parathyroid hormone levels: A population-based study in older men and women. J. Clin. Endocrinol. Metab..

[42] Need A.G., Morris H.A., Horowitz M., Nordin C. (1993). Effects of skin thickness, age, body fat, and sunlight on serum 25-hydroxyvitamin D. Am. J. Clin. Nutr..

[43] Drincic A.T., Armas L.A., Van Diest E.E., Heaney R.P. (2012). Volumetric dilution, rather than sequestration best explains the low vitamin D status of obesity. Obesity.

[44] Drincic A., Fuller E., Heaney R.P., Armas L.A. (2013). 25-Hydroxyvitamin D response to graded vitamin D(3) supplementation among obese adults. J. Clin. Endocrinol. Metab..

[45] Gallagher J.C., Yalamanchili V., Smith L.M. (2013). The effect of vitamin D supplementation on serum 25(OH)D in thin and obese women. J. Steroid Biochem. Mol. Biol..

[46] Holick M.F., Binkley N.C., Bischoff-Ferrari H.A., Gordon C.M., Hanley D.A., Heaney R.P., Murad M.H., Weaver C.M. (2011). Evaluation, treatment, and prevention of vitamin D deficiency: An Endocrine Society clinical practice guideline. J. Clin. Endocrinol. Metab..

[47] Lips P. (2004). Which circulating level of 25-hydroxyvitamin D is appropriate?. J. Steroid Biochem. Mol. Biol..

[48] Vanderschueren D., Pye S.R., O’Neill T.W., Lee D.M., Jans I., Billen J., Gielen E., Laurent M., Claessens F., Adams J.E. (2013). Active vitamin D (1,25-dihydroxyvitamin D) and bone health in middle-aged and elderly men: The European Male Aging Study (EMAS). J. Clin. Endocrinol. Metab..

[49] Lips P. (2006). Vitamin D physiology. Prog. Biophys. Mol. Biol..

[50] Lieben L., Carmeliet G., Masuyama R. (2011). Calcemic actions of vitamin D: Effects on the intestine, kidney and bone. Best Pract. Res. Clin. Endocrinol. Metab..

[51] Shapses S.A., Sukumar D., Burckhardt P., Dawson-Hughes B., Weaver M.C. (2013). The Hormonal Milieu in Obesity and Influences on the Trabecular, Cortical, and Geometric Properties of Bone. Nutritional Influences on Bone Health: 8th International Symposium.

